# A Nitric Oxide-Donor Furoxan Moiety Improves the Efficacy of Edaravone against Early Renal Dysfunction and Injury Evoked by Ischemia/Reperfusion

**DOI:** 10.1155/2015/804659

**Published:** 2015-03-05

**Authors:** Fausto Chiazza, Konstantin Chegaev, Mara Rogazzo, Juan C. Cutrin, Elisa Benetti, Loretta Lazzarato, Roberta Fruttero, Massimo Collino

**Affiliations:** ^1^Dipartimento di Scienza e Tecnologia del Farmaco, Università di Torino, Via P. Giuria 9, 10125 Torino, Italy; ^2^Dipartimento di Biotecnologie Molecolari e Scienze per la Salute, Università di Torino, Via Nizza 52, 10126 Torino, Italy; ^3^ININCA-CONICET, Buenos Aires, Argentina

## Abstract

Edaravone (5-methyl-2-phenyl-2,4-dihydro-3H-pyrazol-3-one, EDV) is a free-radical scavenger reduces organ ischemic injury. Here we investigated whether the protective effects of EDV in renal ischemia/reperfusion (I/R) injury may be enhanced by an EDV derivative bearing a nitric oxide- (NO-) donor furoxan moiety (NO-EDV). Male Wistar rats were subjected to renal ischemia (45 minutes), followed by reperfusion (6 hours). Administration of either EDV (1.2–6–30 *µ*mol/kg, i.v.) or NO-EDV (0.3–1.2–6 *µ*mol/kg, i.v.) dose-dependently attenuated markers of renal dysfunction (serum urea and creatinine, creatinine clearance, urine flow, urinary N-acetyl-*β*-D-glucosaminidase, and neutrophil gelatinase-associated lipocalin/lipocalin-2). NO-EDV exerted protective effects in the dose-range 1.2–6 *µ*mol/kg, while a higher dose (30 *µ*mol/kg) was needed to obtain protection by EDV. Both EDV and NO-EDV modulated tissue markers of oxidative stress and lipid peroxidation. NO-EDV, but not EDV, activated endothelial NO synthase (NOS) and blunted I/R-induced upregulation of inducible NOS, secondary to modulation of Akt and NF-*κ*B activation, respectively. Besides NO-EDV administration inhibited I/R-induced IL-1*β*, IL-18, IL-6, and TNF-*α* overproduction. Overall, these findings demonstrate that the NO-donor moiety contributes to the protection against early renal I/R injury and suggest that NO-donor EDV codrugs are worthy of additional study as innovative pharmacological tools.

## 1. Introduction

**Figure 1:**
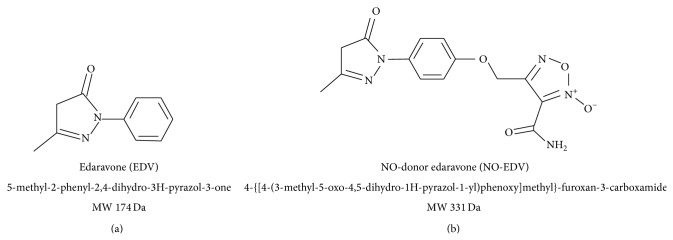
Chemical structures of edaravone (EDV) and edaravone derivative containing NO-donor furoxan moiety (NO-EDV).

Edaravone (5-methyl-2-phenyl-2,4-dihydro-3H-pyrazol-3-one, EDV, Figure 1(a)) is a novel free-radical scavenger, which reacts with free radicals by a one-electron transfer mechanism in polar media, whereas the mechanism of H-atom abstraction has been hypothesized to be predominant in lipid phase [1]. EDV has been approved as neuroprotective agent for the treatment of acute brain infarction by the Japanese Health Organization since 2001 [2]. Moreover, EDV exerts protective effects in patients with acute myocardial infarction [3]. Cases of patients with stroke who unexpectedly developed acute kidney injury (AKI) during EDV administration have been previously reported [4]. However, most recent clinical evidence identifies EDV as a protective factor for the development of AKI [5]. This is in keeping with previous experimental studies showing that EDV exerted protective effects against the occurrence of kidney injury caused by renal ischemia/reperfusion (I/R) [6, 7] and cisplatin administration [8, 9]. Ischemic AKI is an important challenge for clinicians, as it is present in 2% of all hospitalized patients, up to 40% of intensive care unit patients, with a mortality rate >50% in dialyzed patients [10]. Besides, there is currently no successful therapy that can attenuate kidney injury or expedite recovery; thus treatment is only supportive. The etiology of renal ischemic injury is complex and reperfusion, although being essential for the survival of the ischemic kidney, may cause additional injury [11]. Several experimental evidences suggest that nitric oxide (NO) pathway exerts a crucial role in the regulation of renal hemodynamics and function [12]. During ischemia NO protects the ischemic tissue due to its vasodilatatory action. In the reoxygenation phase, NO can react with superoxide radicals, impeding the chain of reaction for additional production of reactive oxygen species (ROS), such as hydrogen peroxide and hydroxyl radical, and thus reducing leukocyte activation and the formation of inflammatory mediators [13]. However, overproduction of NO can be deleterious due to its ability to generate peroxynitrite, which can cause lipid peroxidation of cell membranes.

In this study we investigated the hypothesis that the protective effects of EDV in the early phase of renal I/R may be enhanced by using an EDV derivative bearing a NO-donor furoxan moiety (4-{[4-(3-methyl-5-oxo-4,5-dihydro-1H-pyrazol-1-yl)phenoxy]methyl}-furoxan-3-carboxamide, NO-EDV, Figure 1(b)). NO-EDV has been recently synthesized and characterized by members of our research team within an extended study of NO-donor antioxidant hybrid drugs [14, 15]. The release of NO by NO-EDV was about 14% with respect to the concentration of furoxan after 2 h of incubation with a strong excess of cysteine and studies by electron paramagnetic resonance have demonstrated that the species formed during furoxan ring opening is NO radical. This hybrid compound has been selected for the present study in view of its good balance between antioxidant and vasodilator properties. Specifically, this hybrid drug has been demonstrated to exert potent antioxidant activity, mainly due to the edaravone substructure, and robust vasodilator activity, which parallels that of the corresponding reference simple NO-donor [16]. To gain a better understanding of the effects of EDV in comparison to the related EDV derivative containing the NO-donor function, we carried out further investigation on the role of the NO signaling pathway.

## 2. Materials and Methods

### 2.1. Animals and Surgery

Male Wistar rats (Harlan-Italy; Udine, Italy) were fed with a Piccioni pellet diet (n. 48, Gessate Milanese, Italy) and water* ad libitum*. Animal care was in compliance with Italian regulations on the protection of animals used for experimental and other scientific purposes (D.M. 116/92) as well as the Guide for the Care and Use of Laboratory Animals as adopted and promulgated by the US National Institutes of Health. The renal I/R protocol described here has been approved by the Turin University Ethics Committee and it was employed in multiple previous reports from our laboratory, resulting in significant reproducible and severe (but not fatal) renal dysfunction and injury [17–19]. Briefly, the rats were anesthetized through i.p. injection (30 mg/kg) of Zoletil (15 mg/kg tiletamine + 15 mg/kg zolazepam; Zoletil 100, 100 mg/mL, Laboratoires Virbac, France). The anesthetized rats were placed onto a thermostatically controlled heating pad, a rectal temperature probe was inserted, and body temperature was monitored and maintained at 37°C. A midline laparotomy was performed and the bladder was cannulated for the collection of urine. The kidneys were located and the renal pedicles, containing the renal artery, vein, and nerves, were carefully isolated. The rats were subjected to bilateral renal occlusion for 45 min using nontraumatic artery clamps (Dieffenbach Bulldog Clamps, Harvard Apparatus Ltd., Kent, UK) to clamp the renal pedicles, followed by reperfusion for 6 h. Sham-operated rats underwent identical surgical procedures to those undergoing I/R except that artery clamps were not applied. At the end of the reperfusion, the anaesthetized rats were killed by an overdose of anesthetic, after aortic exsanguination. The kidneys were isolated, weighed, rapidly freeze-clamped with liquid nitrogen, and stored at −80°C until being needed.

### 2.2. Drugs and Treatments

EDV (MW 174 Da) was dissolved in DMSO/propylene glycol/saline solution (5%/20%/75%, resp.) and administered (1.2–6–30 *μ*mol/kg, i.v.) at the beginning of reperfusion and again after 30 min reperfusion. The kinetics and the dose-range were based on those previously shown to ameliorate I/R injury in the rat kidney [20, 21]. NO-EDV (MW 331 Da) was dissolved in DMSO/propylene glycol/saline solution (5%/20%/75%, resp.) and administered (0.3–1.2–6 *μ*mol/kg, i.v.) at the beginning of reperfusion and again after 30 min reperfusion. The NO-EDV dose stoichiometric equivalent to 30 *μ*mol/kg EDV could not be used because of limited solubility of NO-EDV in the vehicle.

Animals were randomly assigned to the following experimental groups:

- sham: rats were treated with the vehicle and subjected to the surgical procedure alone, without causing ischemia (*n* = 8);
- IR: rats were subjected to 45 min ischemia followed by 6 h reperfusion and treated with the vehicle, at the beginning of reperfusion and again after 30 min reperfusion (*n* = 8);
- IR + EDV: rats were subjected to 45 min ischemia followed by 6 h reperfusion and treated with EDV (0.3–1.2–6 *μ*mol/kg), at the beginning of reperfusion and again after 30 min reperfusion (*n* = 8);
- IR + NO-EDV: rats were subjected to 45 min ischemia followed by 6 h reperfusion and treated with NO-EDV (i.v.), at the beginning of reperfusion and again after 30 min reperfusion (*n* = 8).

### 2.3. Measurement of Biochemical Parameters

At the end of the reperfusion period, 1 mL blood samples were collected and centrifuged (10,000 g for 10 min) to separate the serum, from which biochemical parameters were measured within 24 h. The volume of urine produced was determined using the urine collected during the reperfusion period. Serum and urine creatinine concentrations were measured spectrophotometrically at 490 nm by the Jaffé kinetic reaction, using commercially available kits. Renal creatinine clearance was calculated by the standard formula *C* = (*U* × *V*)/*S*, where *U* is the concentration in urine, *V* is urine flow rate, and *S* is the serum concentration. Serum urea and creatinine concentrations and creatinine clearance were used as indicators of impaired renal function. Neutrophil gelatinase associated lipocalin/lipocalin-2 (NGAL) and N-acetyl-*β*-glucosaminidase (NAG) were measured in the urine using commercial enzyme-linked immunosorbent assay (ELISA) kits (MyBiosource, San Diego, CA, USA), and were used as early, sensitive biomarkers for ischemic renal injury [22, 23].

### 2.4. Histopathological Examination and Tissue Injury Scoring

Coronal sections of both kidneys were overnight fixed in 4% buffered formaldehyde solution. Histological examination on PAS stained sections were performed to evaluate the following parameters of tubular cell injury: loss of brush border (LBB: partial or complete loss of the brush border), vacuolar degeneration (vac. deg.: presence of more than three cells with cytoplasmic vacuoles or blebs formation protruding into the lumen of the tubules), tubular dilatation (tub. dil.: dilatation was considered when the tubular lumen was increased at least 20% versus normal corespective), cell detachment (cell det.: presence of necrotic or morphological well preserved isolated cells in the tubular lumen), necrosis (necrosis: presence of three or more cells with signs of coagulative necrosis, such as loss of cell boundaries with marked eosinophilia or extreme swelling together with nuclear changes consisting in pyknosis or chromatolysis or karyorrhexis), and formation of casts (casts: presence of granule-hyaline, mucous or proteinaceous material in the tubular lumen). Five nonconsecutive fields from the cortex and the outer medulla were evaluated (magnification 200x). Renal injury scores were determined by the percentage of tubules involved: 0 = 0; 1 = up to 10%; 2 = 11–20%; 3 = 21–40%; 4 = 41–60%; 5 = more than 61%.

### 2.5. Western Blot Analysis

Western blots were carried out as previously described [24]. Briefly, rat kidney samples were homogenized in 10% homogenization buffer and centrifuged at 1,300 g for 5 minutes at 4°C. Supernatants were removed and centrifuged at 16,000 g at 4°C for 40 minutes to obtain the cytosolic fraction. The pelleted nuclei were resuspended in extraction buffer and centrifuged at 16,000 g for 20 minutes at 4°C. The resulting supernatants containing nuclear proteins were carefully removed, and protein content was determined on both nuclear and cytosolic extracts using a bicinchoninic acid (BCA) protein assay following the manufacturer's directions (Thermo Fisher Scientific, Runcorn, UK). Proteins were separated by 8% sodium dodecyl sulphate-polyacrylamide gel electrophoresis and transferred to polyvinyldenedifluoride membrane, which was then incubated with a primary antibody (rabbit anti-iNOS, mouse anti-phAkt, rabbit anti Akt, goat anti-ph-eNOS, rabbit anti-eNOS, and rabbit anti-MnSOD). Blots were then incubated with a secondary antibody conjugated with horseradish peroxidase (dilution 1 : 10000) and developed using the ECL detection system. The immunoreactive bands were visualized by autoradiography and the density of the bands was evaluated densitometrically using Gel Pro Analyzer 4.5, 2000 software (Media Cybernetics, Silver Spring, MD, USA). The membranes were stripped and incubated with *β*-actin monoclonal antibody (dilution 1 : 5000) and subsequently with an anti-mouse antibody (dilution 1 : 10000) to assess gel-loading homogeneity.

### 2.6. Determination of Malondialdehyde (MDA) Levels

MDA levels were determined in the supernatant fractions of kidney homogenized samples by high-performance liquid chromatography (HPLC) system (Shimadzu VP Class, Shimadzu Corporation, Japan) with fluorescence detection using a commercial reagent kit (Chromsystems Instruments & Chemicals, Gräfelfing, Germany), as previously described [25]. Measurements were expressed in terms of MDA normalized to the tissue protein content.

### 2.7. Determination of Interleukin- (IL-) 1*β*, IL-6, IL-18, and Tumor Necrosis Factor- (TNF-) *α* Production

Cytokines were measured in kidney homogenates using commercial ELISA kits (Cayman Chemical, Ann Arbor, MI), following the protocol provided by the manufacturer.

### 2.8. Materials

Unless otherwise stated, all compounds were purchased from Sigma-Aldrich Company Ltd. (Missouri, USA). The BCA Protein Assay Kit and SuperBlock blocking buffer were from Pierce Biotechnology Inc. (Illinois, USA). Antibodies were from Cell Signaling Technology (Massachusetts, USA). Luminol ECL was from Amersham (Buckinghamshire, UK).

### 2.9. Statistical Analysis

All values in both the text and figures are expressed as mean ± standard error of the mean (S.E.M.) for *n* observations. One-way analysis of variance with Dunnett's posttest was performed using the GraphPad Prism Software (San Diego, California, USA) and *P* values below 0.05 were considered as significant.

## 3. Results

### 3.1. Comparative Effects of Acute Administration of EDV and NO-EDV on I/R-Induced Renal Dysfunction

**Figure 2:**
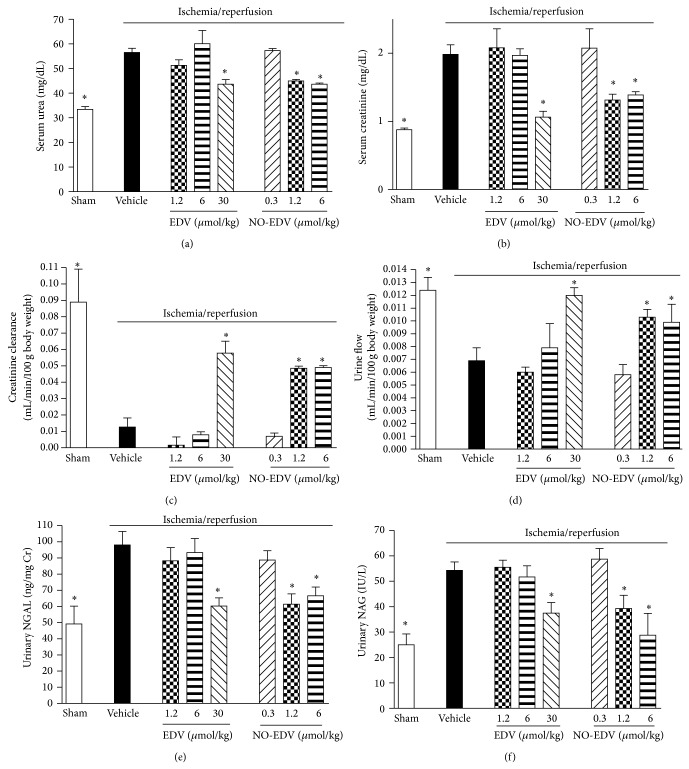
Effects of EDV and NO-EDV on renal dysfunction evaluated on blood and urine parameters. Serum urea (a), creatinine (b), creatinine clearance (c), urine flow (d), and urinary NGAL (e) and NAG (f) levels were measured after sham operation (sham) or renal ischemia-reperfusion injury (vehicle). Further groups of rats received EDV (1.2–30 *μ*mol/kg, i.v.) or NO-EDV (0.3–6 *μ*mol/kg, i.v.) at the beginning of reperfusion and again after 30 min reperfusion. Data are expressed as mean ± S.E.M. ^*^ *P* < 0.05 versus vehicle.

Rats that underwent renal I/R exhibited a significant increase in serum levels of urea and creatinine, compared with sham-operated rats (Figures 2(a) and 2(b), resp.), suggesting a significant degree of renal dysfunction. To discount the possibility of a rapid increase in serum creatinine levels due to increased release of creatinine from muscle during I/R, creatinine clearance was also measured. I/R exposure led to a drastic decrease in creatinine clearance (Figure 2(c)) as well as in urine flow (Figure 2(d)). Administration of EDV significantly attenuated the injury and glomerular dysfunction caused by I/R when tested at the highest dose (30 *μ*mol/kg), with no effects at the doses 1.2 and 6 *μ*mol/kg. Interestingly, NO-EDV evoked a robust improvement in renal function at the doses 1.2 and 6 *μ*mol/kg, which are stoichiometrically equivalent to the noneffective doses of EDV. As shown in Figures 2(e) and 2(f), renal I/R induced a significant increase in urinary NGAL and NAG levels, suggesting significant tubular dysfunction, which was reduced in a dose-dependent way by both EDV and NO-EDV. Notably, whereas EDV was ineffective in the dose-range 1.2 and 6 *μ*mol/kg, the stoichiometric equivalent dose-range of NO-EDV exerted significant protective effects.

### 3.2. Effects of EDV and NO-EDV on the Morphological Alterations Associated to I/R Injury

**Figure 3:**
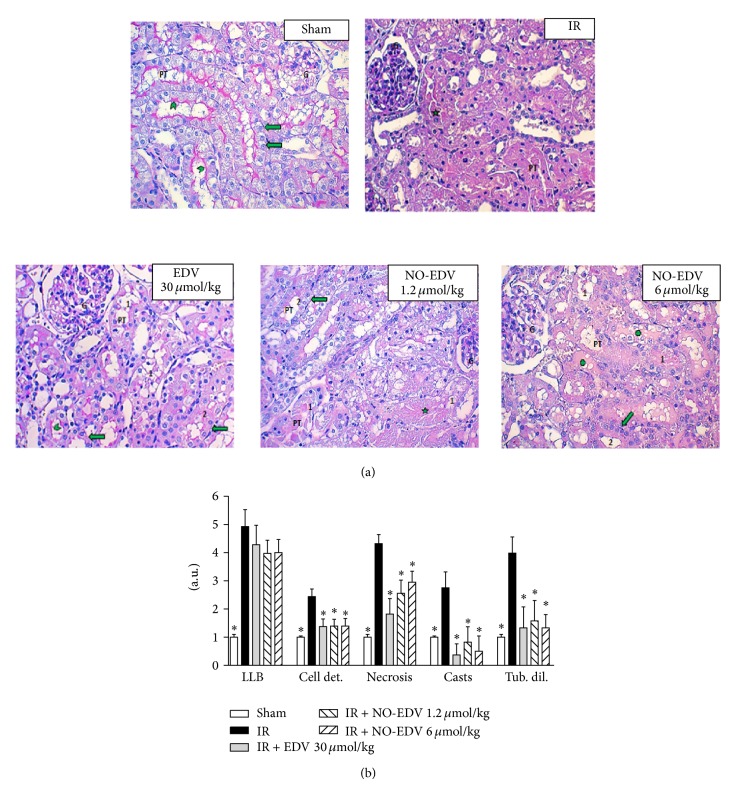
Effects of EDV and NO-EDV on the I/R injured kidney. (a) Representative pictures from the different groups. (b) Semiquantitative assessment of the severity of kidney damage. Histological examination of both kidneys to detect loss of brush border (LBB), cell detachment (cell det.), necrosis (necrosis), formation of casts (casts), and tubular dilatation (tub. dil.) was performed on PAS stained sections. Five nonconsecutive fields from the cortex and the outer medulla were evaluated (magnification 200x). Renal injury scores were determined by the percentage of tubules involved: 0 = 0; 1 = up to 10%; 2 = 11–20%; 3 = 21–40%; 4 = 41–60%, and 5 = more than 61%. G: glomeruli; PT: proximal tubules; green arrowhead: preserved brush border; green arrow: nuclei polarized against the basal membrane; green star: cell debris; green cross indicates hyaline casts. Data are expressed as mean ± S.E.M. ^*^ *P* < 0.01 versus vehicle.

Representative pictures from each experimental group were taken from the transition between the deeper cortex and the outer stripe of the outer medulla zones (Figure 3(a): ×200; PAS stained. G: glomerulus. PT: proximal tubules). When compared against the kidney from the sham group, where the cells of the PT appeared morphologically normal with a well preserved brush border and nuclei polarized against the basal membrane, the major part of the PT from the ischemically injured kidney was mainly composed of coagulated necrotic cells with abundant cell debris occupying the lumen of the tubules. Interestingly after the treatment with EDV (30 *μ*mol/kg) and NO-EDV (6 *μ*mol/kg) the amount of severely damaged and necrotic cells within PTs was significantly decreased. At the same time, many PTs were composed of cells that exhibited a partial preservation of the brush border, as well as the maintenance of the nuclear polarization. The reduction in these renal abnormalities was still evident when animals were treated with NO-EDV at the lowest dose (1.2 *μ*mol/kg). Semiquantitative scoring of kidney injury performed on the histological slides confirmed the visual observations and showed that both EDV and NO-EDV significantly attenuated renal cell damage (Figure 3(b)).

### 3.3. Effects of EDV and NO-EDV on Oxidative Stress Induced by Renal I/R Injury

**Figure 4:**
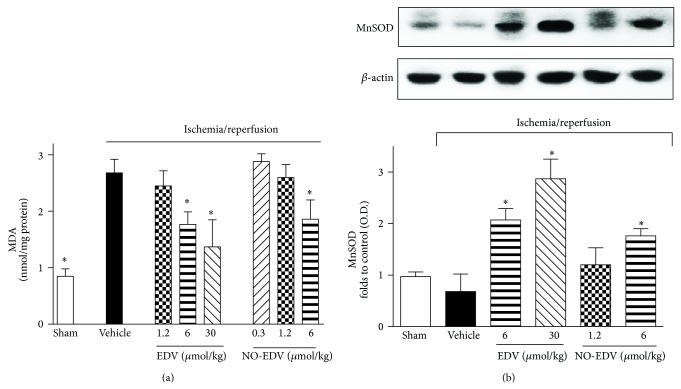
Effects of EDV and NO-EDV on markers of oxidative stress in kidney samples. MDA levels (a) and MnSOD expression (b) were measured subsequent to sham operation (sham) or renal ischemia-reperfusion injury in the absence (vehicle) or presence of EDV (1.2–30 *μ*mol/kg, i.v.) or NO-EDV (0.3–6 *μ*mol/kg, i.v.). Data are expressed as mean ± S.E.M. ^*^ *P* < 0.05 versus vehicle.

Compared to sham-operated rats, kidneys obtained from rats that had undergone I/R exhibited a massive increase in MDA levels, an indicator of lipid peroxidation (Figure 4(a)). The robust increase in lipid peroxidation was blunted by administration of EDV in a dose-dependent manner and partially prevented by NO-EDV at the highest dose tested here (6 *μ*mol/kg). Simultaneously, the expression of the endogenous antioxidant enzyme MnSOD (Figure 4(b)) was upregulated following EDV administration during reperfusion, with maximum effect at 30 *μ*mol/kg. A slight increase in MnSOD expression was also recorded in the group treated with the highest dose of NO-EDV.

### 3.4. Effects of Drug Treatments on Akt and eNOS Phosphorylation in the Kidneys of Rats That Underwent I/R Injury

**Figure 5:**
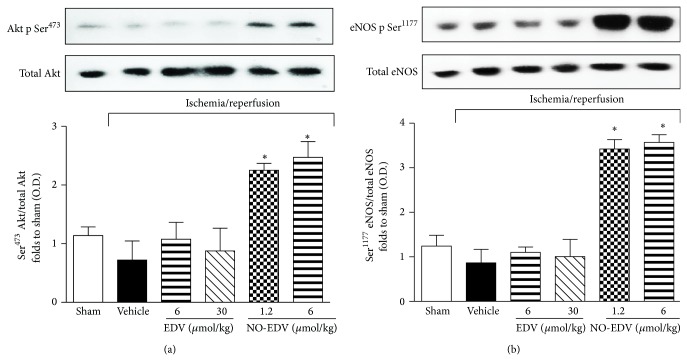
Effects of EDV and NO-EDV on Akt and eNOS phosphorylation. Representative Western blot and corresponding densitometric analysis of the bands showing phosphorylated (Ser^473^) and total Akt (panel (a)) and phosphorylated (Ser^1177^) and total eNOS in rats exposed to I/R in the absence (vehicle) or presence of EDV (6 and 30 *μ*mol/kg, i.v.) or NO-EDV (1.2 and 6 *μ*mol/kg, i.v.). Each immunoblot is from a single experiment and is representative of three separate experiments. Densitometric analysis of the related bands is expressed as relative OD, corrected for the corresponding *β*-actin contents, and normalized using the related sham-operated band. The data from bands densitometric analysis are mean S.E.M. ^*^ *P* < 0.05 versus vehicle.

The degree of the phosphorylation of Akt on Ser^473^ and eNOS on Ser^1177^ in reperfused kidney samples was similar in sham-operated rats and I/R rats, indicating that I/R alone is not sufficient to affect this signaling pathway (Figures 5(a) and 5(b)). Similarly, Akt and eNOS phosphorylation levels were not modified by EDV administration. In contrast, acute administration of NO-EDV (1.2 and 6 *μ*mol/kg) evoked a significant increase in the phosphorylation of Akt and eNOS, suggestive of an increased enzyme activation (Figures 5(a) and 5(b)).

### 3.5. NO-EDV, but Not EDV, Inhibited iNOS Expression, NF-*κ*B Nuclear Translocation, and Cytokine Production Evoked by I/R Injury

**Figure 6:**
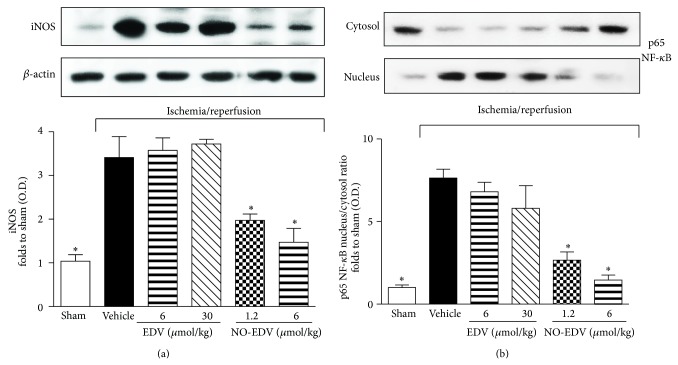
Effects of EDV and NO-EDV on iNOS expression and p65 NF-*κ*B nuclear translocation. Representative Western blot and corresponding densitometric analysis of the bands showing iNOS protein expression (panel (a)) and NF-*κ*B p65 subunit translocation from the cytosol to the nucleus (panel (b)) were evaluated in the kidney homogenates of rats subjected to the surgical procedure alone (sham) or rats subjected to I/R treated with vehicle or EDV (6 and 30 *μ*mol/kg, i.v.) or NO-EDV (1.2 and 6 *μ*mol/kg, i.v.). Each immunoblot is from a single experiment and is representative of three separate experiments. Densitometric analysis of the related bands is expressed as relative OD, corrected for the corresponding *β*-actin contents, and normalized using the related sham-operated band. The data from bands densitometric analysis are mean S.E.M. ^*^ *P* < 0.05 versus vehicle.

**Figure 7:**
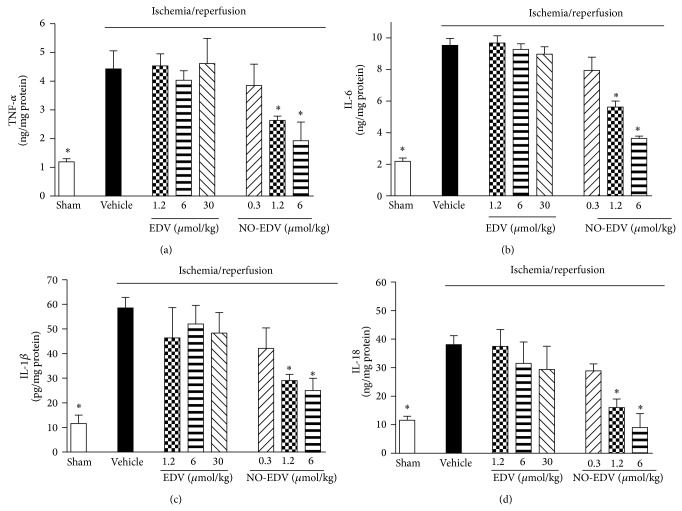
Effects of EDV and NO-EDV on cytokine production in kidney samples. TNF-*α* (a), IL-6 (b), IL-1*β* (c), and IL-18 (d) levels were measured in the kidney of sham-operated rats (sham) and rats that underwent 45 min ischemia and 6 h reperfusion in the absence (vehicle) or presence of EDV (1.2–30 *μ*mol/kg, i.v.) or NO-EDV (0.3–6 *μ*mol/kg, i.v.). Data are mean S.E.M. ^*^ *P* < 0.05 versus vehicle.

When the expression levels of the iNOS isoform were measured (Figure 6(a)), we detected low protein expression in the kidney of sham-operated animals. I/R injury induced a robust increase in the expression of iNOS, which was not further modified by EDV administration. In contrast, rats treated with NO-EDV during reperfusion showed a significant decrease in iNOS overexpression induced by I/R. When compared with sham-operated rats, the kidneys of I/R rats treated with vehicle exhibited significant increases in the nuclear translocation of the p65 NF-*κ*B subunit (Figure 6(b)), indicating the activation of NF-*κ*B. NO-EDV administration resulted in a significant reduction in nuclear translocation of p65 and, hence, in the activation of NF-*κ*B in the kidney. This effect was not recorded when I/R rats were treated with the stoichiometrically equivalent dose of EDV (6 *μ*mol/kg), not even when rats were exposed to the highest dose (30 *μ*mol/kg). TNF-*α*, IL-6, IL-1*β*, and IL-18, typical proinflammatory cytokines, were significantly increased in kidney homogenates from rats exposed to I/R injury, as compared with the sham-operated animals (Figure 7). Interestingly, administration of NO-EDV, but not EDV, reported cytokines concentration back to values similar to those measured in the kidneys of sham-operated animals in a dose-dependent manner.

## 4. Discussion

Our results further corroborate and extend previous findings demonstrating that EDV exerts beneficial effects against organ ischemic damage, including renal I/R [26–30]. Here we showed that acute administration of EDV during reperfusion affected early markers of renal dysfunction and injury evoked by I/R, including reduction in serum creatinine and urea and increases in creatinine clearance and urine flow. Tubular protection is suggested by a significant reduction in urinary excretion of both NAG and NGAL, well-known markers of tubular cell dysfunction. These effects were associated with a significant reduction of local lipid peroxidation as well as an increased expression of the antioxidant enzyme MnSOD. These data are compelling in light of a recent clinical study suggesting that EDV may be a useful medication to protect the kidney function in patients with acute ischemic stroke [5]. Overall, our findings are congruent with recently published data indicating that I/R-induced kidney damage occurs very early, already during the first hours of reperfusion injury, and can be significantly alleviated by an early intervention, which not only can be beneficial in terms of the acute alterations but may also have positive impact on the long-term effects of renal damage [31–33].

Although the molecular mechanisms underlying the I/R-induced renal injury are yet poorly understood, several causal factors (including ATP depletion, ROS production, and leukocyte activation) have been shown to contribute to its pathogenesis [34]. A key role in the homeostasis of the renal hemodynamic and function is exerted by the soluble gas NO. Decreased NO bioavailability is accompanied by endothelial cell dysfunction and impaired renal microcirculation [35, 36] and changes in NO production in the kidney are closely related to various pathological conditions, such as chronic renal failure, lipopolysaccharide-induced renal dysfunction, and AKI [37, 38]. Accordingly, several studies have demonstrated that endogenous or exogenous NO protects the kidney against I/R injury [39–41]. In keeping with these evidences, we demonstrated here for the first time that the acute administration of an EDV derivative containing NO-donor function caused a pronounced reduction in early markers of renal I/R injury and dysfunction, similar to the one afforded by EDV. The limited solubility of NO-EDV in the vehicle did not allow a direct comparison at the same stoichiometric equivalent highest dose (30 *μ*mol/kg). Nevertheless, it is noteworthy that protective effects by NO-EDV were detected in the dose-range 1.2–6 *μ*mol/kg, while the stoichiometric equivalent doses of EDV showed no effect and a higher dose (30 *μ*mol/kg) was needed to obtain protection by EDV. Thus, it appears that the NO-donor moiety significantly contributes to counteract the renal I/R injury, leading to obtaining protective effects at doses lower than those required by EDV alone. This increased efficacy is due, at least in part, to the activation of additive protective intracellular mechanisms by the NO-donor substructure. For instance, NO-EDV caused a robust increase in phosphorylation of specific serine residues in Akt, which was not recorded when animals were treated with EDV. Akt is a member of the phosphoinositide 3-kinase signal transduction enzyme family which, upon phosphorylation by its upstream regulator, can promote eNOS activation and exert several anti-inflammatory and antiapoptotic effects [42]. A reduction in the activation of this important survival pathway has been recently demonstrated to make the kidney more susceptive to I/R insult [43, 44]. Here we show that the increase in Akt phosphorylation was associated with massive eNOS, activation, thus resulting in an enhanced formation of NO in the microcirculation. The enhanced activation of eNOS (possibly secondary to activation of Akt) should make the kidney of animals treated with NO-EDV more resistant to injury. Our results are corroborated by other studies demonstrating that NO generated by constitutive eNOS is beneficial and mechanisms that increase eNOS activity protect against renal I/R injury [45–48]. In contrast, the role of iNOS in renal I/R injury remains controversial. Some studies have demonstrated that renal I/R injury was efficiently attenuated by genetic deficiency or pharmacological blockade of iNOS [49–51], while other studies clearly demonstrated that iNOS-generated NO inhibited ischemic renal damage [52–54]. This difference may be due to the different levels of NO production associated with the degree or method of I/R injury. We demonstrated here that NO-EDV significantly enhanced eNOS activation and this effect was associated with reduced iNOS expression in the kidney of rat that underwent I/R injury in comparison to vehicle-treated animals. A basal production of NO is necessary for maintaining an adequate glomerular function, since the inhibition of NO synthesis may increase both efferent glomerular arteriolar resistance and glomerular capillary pressure and induce significant changes in renal histology [55]. It is noteworthy that the NO-donor moiety is linked to the radical scavenger EDV which can further prevent the reaction of NO with the superoxide radical, impeding the chain of reaction for additional production of ROS such as hydroxyl radical and hydrogen peroxide, which are known to contribute to kidney structural and functional alteration [56]. An additional contribution to the regulatory effects of NO-EDV on NO pathway may rely on the well-known NO ability to inhibit the transcription factor NF-*κ*B activity via several mechanisms [57, 58]. In the present study we documented a reduced NF-*κ*B nuclear translocation in the presence of NO-EDV. As iNOS expression is mainly regulated by NF-*κ*B [59] and it is attractive to speculate that NO-EDV might ultimately inhibit I/R-induced iNOS overexpression throughout a negative feedback-like mechanism. NF-*κ*B plays also an important role in regulating the transcription of several other genes, especially those involved in producing mediators of local and systemic inflammation, such as cytokines and chemokines. Thus, the reduced activation of this transcription factor by NO-EDV may also account for the decreased production of cytokines in the kidney of animals that underwent I/R injury. Overall, these effects on selective signaling pathways may help to explain the lower dose-range by NO-EDV in comparison with EDV needed to protect the kidney against I/R injury. Unfortunately, the lack of direct comparison between NO-EDV and a pharmacological strategy relying exclusively on NO supplementation does not allow us to offer ultimate evidence on the specific contribution by the NO-donor substructure.

Finally, as AKI has been described as an acute inflammatory disease with chronic effects [60], the early inhibition of inflammatory signaling pathways and related cytokine production by NO-EDV might account for long-term beneficial effects superior to those achieved by a sole antioxidant strategy. This is in keeping with recent evidence showing that therapeutic strategies ameliorating endothelial cell dysfunction and cell infiltration reduce the progression to renal interstitial fibrosis, a characteristic pathologic change of chronic kidney disease, developing several days after the ischemic injury [61]. It has to be stressed, however, that the lack of long-term evaluation in our study limits this interpretation and the clinical transferability of our findings. Thus, a further rigorous evaluation of the effects of the tested compound against the functional and histological abnormalities detectable at longer reperfusion times are warranted to confirm and better quantify the NO-donor moiety contribution.

## 5. Conclusions

In conclusion, we confirmed that EDV exerts protective effects in an early stage of renal I/R injury and, most notably, we demonstrated for the first time that the NO-donor moiety improves EDV efficacy by evoking anti-inflammatory and endothelial protective effects throughout the modulation of selective signaling pathways. Overall, our experiments suggest that the administration of a hybrid compound with both NO-donor and EDV-related antioxidant properties may represent a novel pharmacological strategy against I/R injury. Nevertheless, further data are warranted to assess the stability performance, long-term effects, safety, and cost requirements of these hybrid compounds, which can significantly affect the robustness of the pharmacological approach proposed here.

## Abbreviations

EDV: Edaravone
AKI: Acute kidney injury
I/R: Ischemia/reperfusion
NO: Nitric oxide
NGAL: Neutrophil gelatinase associated lipocalin/lipocalin-2
NAG: N-Acetyl-*β*-glucosaminidase
TBARS: Thiobarbituric acid-reactive substances
eNOS: Endothelial nitric oxide synthase
iNOS: Inducible nitric oxide synthase.

## References

[1] Pérez-González A., Galano A. (2011). OH radical scavenging activity of edaravone: mechanism and kinetics. *Journal of Physical Chemistry B*.

[2] Toyoda K., Fujii K., Kamouchi M. (2004). Free radical scavenger, edaravone, in stroke with internal carotid artery occlusion. *Journal of the Neurological Sciences*.

[3] Tsujita K., Shimomura H., Kawano H. (2004). Effects of edaravone on reperfusion injury in patients with acute myocardial infarction. *The American Journal of Cardiology*.

[4] Watanabe T., Tahara M., Todo S. (2008). The novel antioxidant edaravone: from bench to bedside. *Cardiovascular Therapeutics*.

[5] Kamouchi M., Sakai H., Kiyohara Y., Minematsu K., Hayashi K., Kitazono T. (2013). Acute kidney injury and edaravone in acute ischemic stroke: the fukuoka stroke registry. *Journal of Stroke and Cerebrovascular Diseases*.

[6] Tahara M., Nakayama M., Jin M. B. (2005). A radical scavenger, edaravone, protects canine kidneys from ischemia-reperfusion injury after 72 hours of cold preservation and autotransplantation. *Transplantation*.

[7] Matsuyama M., Hayama T., Funao K. (2006). Treatment with edaravone improves the survival rate in renal warm ischemia-reperfusion injury using rat model. *Transplantation Proceedings*.

[8] Sueishi K., Mishima K., Makino K. (2002). Protection by a radical scavenger edaravone against cisplatin-induced nephrotoxicity in rats. *European Journal of Pharmacology*.

[9] Iguchi T., Nishikawa M., Chang B. (2004). Edaravone inhibits acute renal injury and cyst formation in cisplatin-treated rat kidney. *Free Radical Research*.

[10] Bellomo R., Kellum J. A., Ronco C. (2012). Acute kidney injury. *The Lancet*.

[11] Martins P. N. A., Chandraker A., Tullius S. G. (2006). Modifying graft immunogenicity and immune response prior to transplantation: potential clinical applications of donor and graft treatment. *Transplant International*.

[12] Majid D. S. A., Navar L. G. (2001). Nitric oxide in the control of renal hemodynamics and excretory function. *The American Journal of Hypertension*.

[13] Rhoden E. L., Rhoden C. R., Lucas M. L., Pereira-Lima L., Zettler C., Belló-Klein A. (2002). The role of nitric oxide pathway in the renal ischemia-reperfusion injury in rats. *Transplant Immunology*.

[14] Chegaev K., Lazzarato L., Rolando B. (2007). NO-donor melatonin derivatives: synthesis and in vitro pharmacological characterization. *Journal of Pineal Research*.

[15] Cena C., Chegaev K., Balbo S. (2008). Novel antioxidant agents deriving from molecular combination of vitamin C and NO-donor moieties. *Bioorganic and Medicinal Chemistry*.

[16] Chegaev K., Cena C., Giorgis M. (2009). Edaravone derivatives containing NO-donor functions. *Journal of Medicinal Chemistry*.

[17] Collino M., Benetti E., Miglio G. (2011). Peroxisome proliferator-activated receptor *β*/*δ* agonism protects the kidney against ischemia/reperfusion injury in diabetic rats. *Free Radical Biology and Medicine*.

[18] Patel N. S. A., Cuzzocrea S., Collino M. (2007). The role of cycloxygenase-2 in the rodent kidney following ischaemia/reperfusion injury in vivo. *European Journal of Pharmacology*.

[19] Collino M., Rogazzo M., Pini A. (2013). Acute treatment with relaxin protects the kidney against ischaemia/reperfusion injury. *Journal of Cellular and Molecular Medicine*.

[20] Doi K., Suzuki Y., Nakao A., Fujita T., Noiri E. (2004). Radical scavenger edaravone developed for clinical use ameliorates ischemia/reperfusion injury in rat kidney. *Kidney International*.

[21] Li Y., Xia A.-Z., Xing S.-H. (2010). Protective effect of edaravone against renal ischemia/reperfusion injury and compared with ischemic postconditioning in rats. *Yaoxue Xuebao*.

[22] Bazzi C., Petrini C., Rizza V. (2002). Urinary *N*-acetyl-*β*-glucosaminidase excretion is a marker of tubular cell dysfunction and a predictor of outcome in primary glomerulonephritis. *Nephrology Dialysis Transplantation*.

[23] Mishra J., Qing M. A., Prada A. (2003). Identification of neutrophil gelatinase-associated lipocalin as a novel early urinary biomarker for ischemic renal injury. *Journal of the American Society of Nephrology*.

[24] Patel N. S. A., Kerr-Peterson H. L., Brines M. (2012). Delayed administration of pyroglutamate helix B surface peptide (pHBSP), a novel nonerythropoietic analog of erythropoietin, attenuates acute kidney injury. *Molecular Medicine*.

[25] Kenan Kinaci M., Erkasap N., Kucuk A., Koken T., Tosun M. (2012). Effects of quercetin on apoptosis, NF-kappaB and NOS gene expression in renal ischemia/reperfusion injury. *Experimental and Therapeutic Medicine*.

[26] Zhang N., Komine-Kobayashi M., Tanaka R., Liu M., Mizuno Y., Urabe T. (2005). Edaravone reduces early accumulation of oxidative products and sequential inflammatory responses after transient focal ischemia in mice brain. *Stroke*.

[27] Higashi Y., Jitsuiki D., Chayama K., Yoshizumi M. (2006). Edaravone (3-methyl-1-phenyl-2-pyrazolin-5-one), a novel free radical scavenger, for treatment of cardiovascular diseases. *Recent Patents on Cardiovascular Drug Discovery*.

[28] Tamamura M., Saito M., Kinoshita Y. (2010). Protective effect of edaravone, a free-radical scavenger, on ischaemia-reperfusion injury in the rat testis. *BJU International*.

[29] Shimoda M., Iwasaki Y., Okada T., Kubota K. (2012). Edaravone inhibits apoptosis caused by ischemia/ reperfusion injury in a porcine hepatectomy model. *World Journal of Gastroenterology*.

[30] Kara M., Daglioglu Y. K., Kuyucu Y., Tuli A., Tap O. (2012). The effect of edaravone on ischemia-reperfusion injury in rat ovary. *European Journal of Obstetrics Gynecology and Reproductive Biology*.

[31] Urbschat A., Zacharowski K., Obermüller N. (2014). The small fibrinopeptide B*β*15-42 as renoprotective agent preserving the endothelial and vascular integrity in early ischemia reperfusion injury in the mouse kidney. *PLoS ONE*.

[32] Bellinger M. A., Bean J. S., Rader M. A. (2014). Concordant changes of plasma and kidney microRNA in the early stages of acute kidney injury: time course in a mouse model of bilateral renal ischemia-reperfusion. *PLoS ONE*.

[33] di Paola R., Impellizzeri D., Mondello P. (2012). Palmitoylethanolamide reduces early renal dysfunction and injury caused by experimental ischemia and reperfusion in mice. *Shock*.

[34] Edelstein C. L., Ling H., Schrier R. W. (1997). The nature of renal cell injury. *Kidney International*.

[35] Clapp B. R., Hingorani A. D., Kharbanda R. K. (2004). Inflammation-induced endothelial dysfunction involves reduced nitric oxide bioavailability and increased oxidant stress. *Cardiovascular Research*.

[36] Ogita H., Liao J. K. (2004). Endothelial function and oxidative stress. *Endothelium*.

[37] Caramelo C., Espinosa G., Manzarbeitia F. (1996). Role of endothelium-related mechanisms in the pathophysiology of renal ischemia/reperfusion in normal rabbits. *Circulation Research*.

[38] Schwartz D., Mendonca M., Schwartz I. (1997). Inhibition of constitutive nitric oxide synthase (NOS) by nitric oxide generated by inducible NOS after lipopolysaccharide administration provokes renal dysfunction in rats. *The Journal of Clinical Investigation*.

[39] Schramm L., Heidbreder E., Schmitt A. (1994). Role of L-arginine-derived NO in ischemic acute renal failure in the rat. *Renal Failure*.

[40] Linas S., Whittenburg D., Repine J. E. (1997). Nitric oxide prevents neutrophil-mediated acute renal failure. *The American Journal of Physiology—Renal Physiology*.

[41] Tripatara P., Patel N. S. A., Webb A. (2007). Nitrite-derived nitric oxide protects the rat kidney against ischemia/reperfusion injury in vivo: role for xanthine oxidoreductase. *Journal of the American Society of Nephrology*.

[42] Cantley L. C. (2002). The phosphoinositide 3-kinase pathway. *Science*.

[43] El Eter E. A., Aldrees A. (2012). Inhibition of proinflammatory cytokines by SCH79797, a selective protease-activated receptor 1 antagonist, protects rat kidney against ischemia-reperfusion injury. *Shock*.

[44] Jang H.-S., Kim J., Kim K. Y., Kim J. I., Cho M. H., Park K. M. (2012). Previous ischemia and reperfusion injury results in resistance of the kidney against subsequent ischemia and reperfusion insult in mice; A role for the Akt signal pathway. *Nephrology Dialysis Transplantation*.

[45] Choi D. E., Jeong J. Y., Lim B. J. (2009). Pretreatment of sildenafil attenuates ischemia-reperfusion renal injury in rats. *Renal Physiology—American Journal of Physiology*.

[46] Satake A., Takaoka M., Nishikawa M. (2008). Protective effect of 17*β*-estradiol on ischemic acute renal failure through the PI3K/Akt/eNOS pathway. *Kidney International*.

[47] Liu X., Chen H., Zhan B. (2007). Attenuation of reperfusion injury by renal ischemic postconditioning: the role of NO. *Biochemical and Biophysical Research Communications*.

[48] Hussein A.-A. M., Barakat N., Awadalla A., Shokeir A. A. (2012). Systemic and renal haemodynamic changes in renal schemia/reperfusion injury: impact of erythropoietin. *Canadian Journal of Physiology and Pharmacology*.

[49] Chatterjee P. K., Patel N. S. A., Kvale E. O. (2002). Inhibition of inducible nitric oxide synthase reduces renal ischemia/reperfusion injury. *Kidney International*.

[50] Donnahoo K. K., Shames B. D., Harken A. H., Meldrum D. R. (1999). The role of tumor necrosis factor in renal ischemia-reperfusion injury. *The Journal of Urology*.

[51] Walker L. M., Walker P. D., Imam S. Z., Ali S. F., Mayeux P. R. (2000). Evidence for peroxynitrite formation in renal ischemia-reperfusion injury: studies with the inducible nitric oxide synthase inhibitor L-N6-(1-iminoethyl)lysine. *Journal of Pharmacology and Experimental Therapeutics*.

[52] Park K. M., Byun J.-Y., Kramers C., Kim J. I., Huang P. L., Bonventre J. V. (2003). Inducible nitric-oxide synthase is an important contributor to prolonged protective effects of ischemic preconditioning in the mouse kidney. *The Journal of Biological Chemistry*.

[53] Torras J., Herrero-Fresneda I., Lloberas N., Riera M., Cruzaoo J. M., Grinyo J. M. (2002). Promising effects of ischemic preconditioning in renal transplantation. *Kidney International*.

[54] Kim J. I., Jang H.-S., Park K. M. (2010). Endotoxin-induced renal tolerance against ischemia and reperfusion injury is removed by iNOS, but not eNOS, gene-deletion. *BMB Reports*.

[55] Goligorsky M. S., Brodsky S. V., Noiri E. (2002). Nitric oxide in acute renal failure: NOS versus NOS. *Kidney International*.

[56] Paller M. S., Hoidal J. R., Ferris T. F. (1984). Oxygen free radicals in ischemic acute renal failure in the rat. *The Journal of Clinical Investigation*.

[57] Peng H.-B., Libby P., Liao J. K. (1995). Induction and stabilization of I kappa B alpha by nitric oxide mediates inhibition of NF-kappa B. *The Journal of Biological Chemistry*.

[58] Matthews J. R., Botting C. H., Panico M., Morris H. R., Hay R. T. (1996). Inhibition of NF-*κ*B DNA binding by nitric oxide. *Nucleic Acids Research*.

[59] Dijkstra G., Moshage H., Jansen P. L. M. (2002). Blockade of NF-*κ*B activation and donation of nitric oxide: new treatment options in inflammatory bowel disease?. *Scandinavian Journal of Gastroenterology, Supplement*.

[60] Bonventre J. V., Zuk A. (2004). Ischemic acute renal failure: an inflammatory disease?. *Kidney International*.

[61] Furuichi K., Kaneko S., Wada T. (2009). Chemokine/chemokine receptor-mediated inflammation regulates pathologic changes from acute kidney injury to chronic kidney disease. *Clinical and Experimental Nephrology*.

